# Creating a Parent-Informed Pediatric Emergency Department Wait Time App: Human-Centered Design Approach to Creating an AI Health Care Tool

**DOI:** 10.2196/66644

**Published:** 2025-08-29

**Authors:** Sasha Litwin, Maya Mohabir, Ipek Sevim Kocak, Devin Singh

**Affiliations:** 1 Department of Pediatrics, Faculty of Medicine, University of Toronto, Medical Sciences Building, 1 King's College Circle, Toronto, ON, M5S 1A8, Canada, 1 4164009248; 2 Department of Social Science, University of Western Ontario, London, ON, Canada; 3 Department of Physiology and Pharmacology, University of Western Ontario, London, ON, Canada

**Keywords:** emergency department, emergency room, wait times, human-centered design, design thinking, co-design, machine learning, artificial intelligence, algorithm, model, analytics, mHealth, mobile app, smartphone, digital health, digital technology, digital intervention

## Abstract

**Background:**

Waiting has become an unfortunate reality for parents seeking care for their child in the emergency department (ED). Long wait times are known to increase morbidity and mortality. Providing patients with information about their wait time increases their satisfaction and sense of control. There are very few patient-facing artificial intelligence (AI) tools currently in use in EDs, particularly tools that are co-designed with patients and caregivers.

**Objective:**

The aim of this study is to use insights from parents and health care providers to inform the design of an AI tool that provides personalized wait time and health information to parents during their child’s ED visit.

**Methods:**

The study followed a human-centered design methodology. The study was conducted in a large urban tertiary care academic children’s hospital. Data were collected through demographic surveys, semistructured interviews, card sorting, structured observations, and prototype testing with parents and triage nurses. Quantitative data from demographic surveys and card sorting were analyzed using descriptive statistics, including means, medians, and interquartile ranges. Qualitative data from semistructured interviews and observations were analyzed using a thematic analysis. The thematic analysis informed the design criteria of the tool. The tool was implemented in the ED and improved through iterative rounds of usability testing.

**Results:**

Between May 30, 2023, and August 30, 2023, a total of 64 semistructured interviews were conducted with parents in the waiting room. Five interviews were conducted with triage nurses. Parents primarily were mothers (38/64, 59%), were college/university graduates (37/64, 58%), and had a preferred language of English (51/64, 80%). All parents had a smartphone and 97% (62/64) used apps on their smartphone. Children were a median of 7 years old (IQR 4‐12 years old) and had a median of 4 lifetime visits to the ED (IQR 1 to >5). The thematic analysis revealed 5 key themes that informed the development of the tool: (1) anxiety due to uncertainty, (2) feeling forgotten, (3) low health literacy, (4) not understanding the ED process, and (5) no indication of progress.

**Conclusions:**

This study used a human-centered design approach to explore parents’ experience waiting in the pediatric ED to develop an AI tool to improve the waiting experience. By prioritizing parents’ experiences and insights, we created a solution that addresses the challenges of communicating wait times and contributes to a more compassionate and efficient health care environment. The implementation of this tool has given patients and families the control and certainty they were lacking by providing information about their wait time. Successful implementation of technology in health care requires a design approach so that solutions are clinically relevant, user-centered, and tested for acceptability and usability.

## Introduction

Waiting has become an unfortunate reality of seeking care in the emergency department (ED), particularly for parents of pediatric patients [1]. Prolonged wait times in the ED are associated with increased morbidity and mortality in both pediatric and adult populations [2-4]. For parents, the waiting period presents numerous challenges, often marked by stress, anxiety, and uncertainty. When delays occur without accompanying information or updates, the experience can be especially frustrating and psychologically distressing [5]. The absence of communication regarding care timelines and expectations can intensify feelings of helplessness and, in some instances, contribute to aggressive or violent behavior directed at health care personnel [6]. Moreover, inadequate communication and expectations about the ED process can negatively affect the overall family experience and compromise the quality of care delivered to the child [7].

Despite these known challenges, a significant gap remains in how EDs manage and communicate wait times. In many settings, triage nurses are responsible for verbally updating families about delays, often interrupting their clinical duties to do so. Some EDs use static signage in waiting areas to communicate generalized information; however, this is frequently insufficient or inaccessible to patients [8]. In recent years, certain EDs have introduced digital wait time displays in the department to enhance transparency [9]. Some institutions have made wait time estimates available through websites or mobile apps, allowing access from both the ED and home [5].

The provision of wait time information has been shown to improve patient satisfaction and enhance individuals’ perceived sense of control while in the ED [10]. Emerging patient-facing technologies, particularly those leveraging artificial intelligence (AI), offer an opportunity to address this unmet need. AI-based tools can generate personalized wait time predictions tailored to individual patients based on their clinical presentation and dynamic departmental factors, rather than providing a single generic estimate [11]. Such tools have the potential to enhance the experience of care, reduce patient frustration, and minimize interruptions to clinical workflows. By proactively setting expectations and offering transparency, personalized wait time information may empower families and decrease the incidence of disruptive behavior toward staff, allowing providers to focus on critical medical tasks.

Over the past decade, there has been a growing emphasis on patient-centered frameworks in health care delivery [12]. Engaging patients and families in the design and development of health technologies can lead to more effective, acceptable, and implementable solutions [13,14]. Human-centered design methodology emphasizes collaboration with end users, such as patients, parents, and health care workers, to ensure health care tools are informed by their needs, preferences, and lived experiences [12]. Nevertheless, there is a lack of research outlining best practices for applying patient-centered design principles to the development of advanced digital tools, such as AI applications, in clinical settings.

Given the known impact of wait time communication on patient experience and the importance of incorporating patient and caregiver perspectives into the design of health care technologies, the objective of this study was to use insights from patients, caregivers, and health care providers to inform the development of a patient-facing AI tool. The AI tool aims to provide parents with personalized wait time estimates and health education resources during their child’s ED visit.

## Methods

### Design

The study followed a human-centered design approach, which is a prospective, iterative, and participatory approach to research and problem solving [15,16]. We used several design and qualitative research methods to gather and analyze data (Table 1 shows the techniques used).

### Ethical Considerations

The study was reviewed and approved by the hospital’s Quality and Risk Management Office and was therefore exempt from review by the research ethics board. All participants were provided with verbal and written information about the study prior to enrollment. Verbal informed consent was obtained from all participants before participation. The study adhered to institutional guidelines for informed consent and complied with local, national, and international regulations on the protection of personal information, privacy, and human rights. No financial compensation or stipend was provided to participants.

**Table 1.** Design research techniques.

Technique used	Description	Participants involved
Semistructured interviews	The first round of interviews focused on the waiting experience, other waiting environments, and a card-sorting activity. The second round of interviews focused on unmet needs while waiting and prototype testing. Interviews with triage nurses focused on commonly asked questions from parents in the waiting room.	64 parents (40 during round 1, 24 during round 2) and 5 triage nurses
Card sorting	Participants were given 8 cards with different data points and were instructed to sort the cards from most important to least important, for example, “How many children are waiting ahead of my child?” [17]	31 parents
Prototype testing	Prototype testing allows designers to gather feedback on a low-fidelity version of the product [18,19]. Prototype testing occurred during the second round of semistructured interviews. Parents were shown prototypes of the tool on a tablet device (Apple iPad) and asked for their feedback with structured questions.	24 parents
Structured observations	Structured observations using an AEIOU^a^ Observation Tool (Multimedia Appendix 1) were conducted in the waiting room during two time periods by research assistants. Structured observations allow the research team to analyze the people, objects, and interactions in the space [20].	2 observation periods

a AEIOU: Activities, Environments, Interactions, Objects, Users.

### Setting

The study was conducted in an academic tertiary care children’s hospital that cares for patients from birth to 18 years of age. The hospital is in Toronto, Canada, a large urban area notable for its cultural, racial, language, and socioeconomic diversity. The annual census for the ED is approximately 90,000 patients per year. Interviews and observations were conducted in the waiting room of the ED.

### Recruitment

Data were collected between May 30, 2023, and August 30, 2023. Participants were selected by convenience sampling. Participants were included if their child was waiting to be seen in the ED. Parents of unstable or acutely ill children (based on the triage nurse assessment) were excluded. Participants were approached to participate by one of two research assistants on the study team. Research assistants approached families who were in the waiting room with their child.

Two female research assistants conducted the interviews and observations. The research assistants were both undergraduate students with an interest in clinical research. The research assistants did not have any relationship to the participants prior to commencing the study.

### Instrument Development

A semistructured interview guide was created by the research team (DS, SL, MM, ISK) after a review of relevant literature and consultation with subject matter experts (Multimedia Appendix 1). Interview guides were pretested with 3 research assistants who were not involved in the study and pilot-tested with 2 parents from the sample population. The research assistants further refined the interview script by conducting mock interviews with experienced research assistants prior to the start of the study.

### Data Collection

Data were collected through demographic surveys, semistructured interviews, card sorting [17], structured observations [20], and prototype testing [18,19] (see Table 1 for techniques used).

Interviews and observations were conducted by research assistants while participants were in the waiting room of the ED. When the waiting room was crowded, research assistants brought participants to a nearby examination room for more privacy. Interviews lasted 20‐45 minutes each. Interviews were conducted with participants verbally, with one research assistant asking the question and a second research assistant typing the participant’s answers verbatim into a secure online form (Microsoft Forms). Research assistants recorded field notes and comments in the online form. No audio or video recordings were collected. Interviews were conducted in the participant’s preferred language, using a telephone interpretation service when needed.

**Figure 1.**
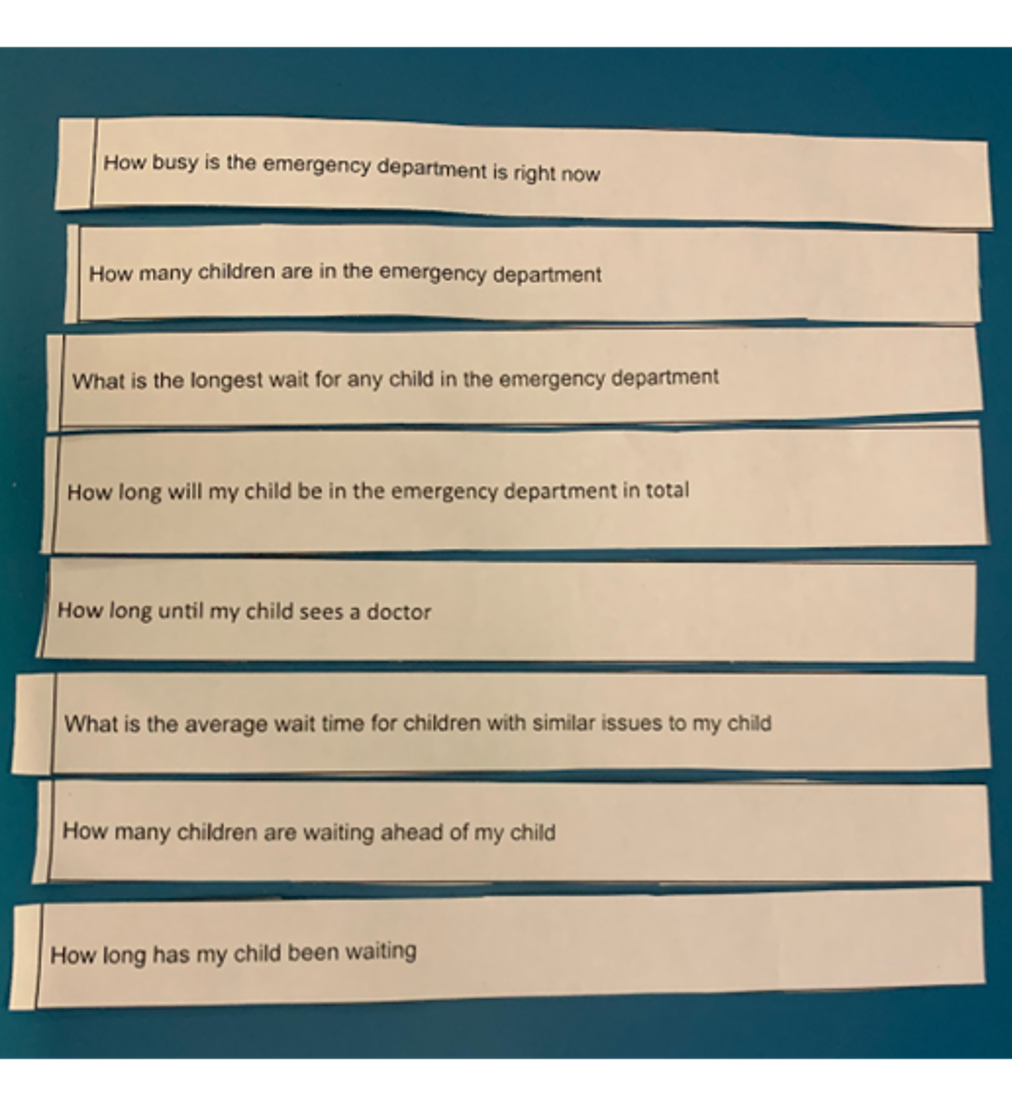
Card sorting example.

**Figure 2.**
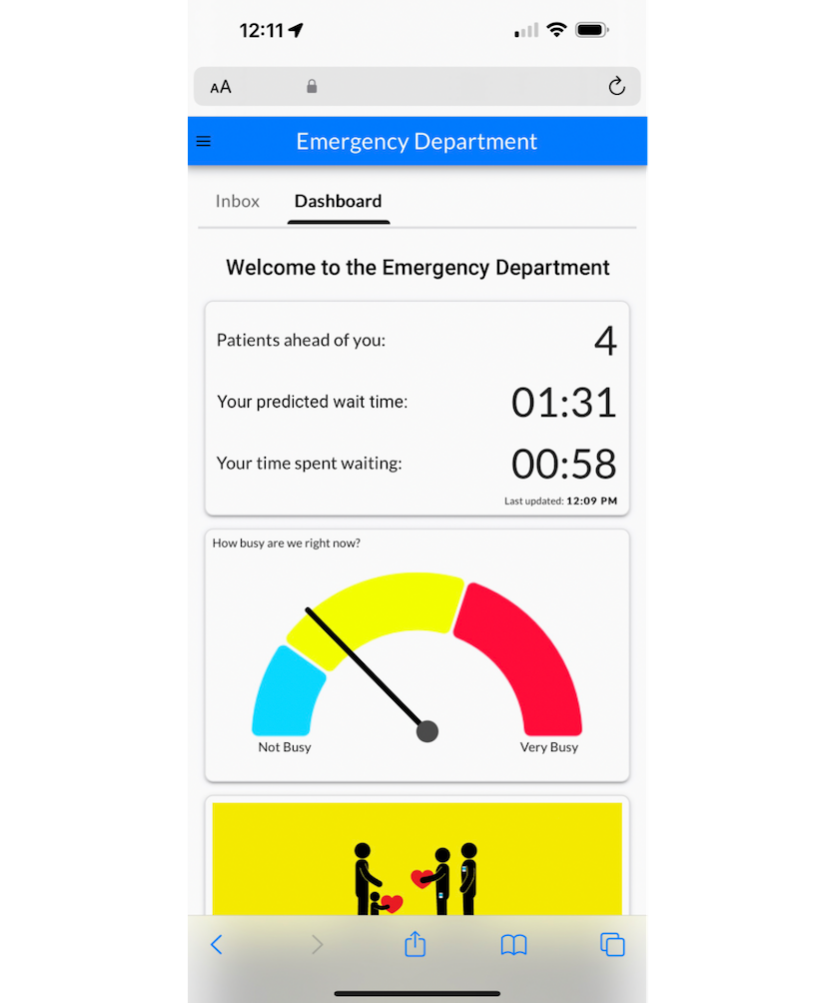
Example prototype.

Two rounds of semistructured interviews were conducted with parents. The first round focused on the waiting experience in the ED; other waiting experiences with their child, such as at a restaurant, the airport, amusement park, or service center; and a card-sorting activity (see Figure 1 for an example of card sorting). The second round of interviews focused on parents’ unmet needs while waiting and prototype testing. Parents were shown wireframe prototypes of the wait time app on a tablet device (Apple iPad) and asked for their feedback with structured questions (see Figure 2 for examples of prototypes). In addition, brief (5‐10 min) intercept interviews were conducted with triage nurses to corroborate commonly asked questions from parents in the waiting room. Both research assistants conducted one observation session in the waiting room. Observations were recorded on a structured observation chart (Multimedia Appendix 2).

The sample size was guided by the principle of data saturation, where participants were recruited until no new codes or themes emerged from the data. Although qualitative research does not require statistical calculations for sample size, an estimated range of 12‐20 participants was initially planned based on similar literature and the scope of the research question [21]. Given the iterative nature of thematic analysis, data collection and analysis occurred concurrently, allowing for ongoing assessment of saturation. Recruitment ceased when additional data no longer contributed novel insights to the thematic framework.

### Data Analysis

Quantitative data from demographic surveys and card sorting were analyzed using descriptive statistics, including means, medians, and interquartile ranges. Data were not extracted or analyzed from the wait time app as the software was in development during the study.

**Figure 3.**
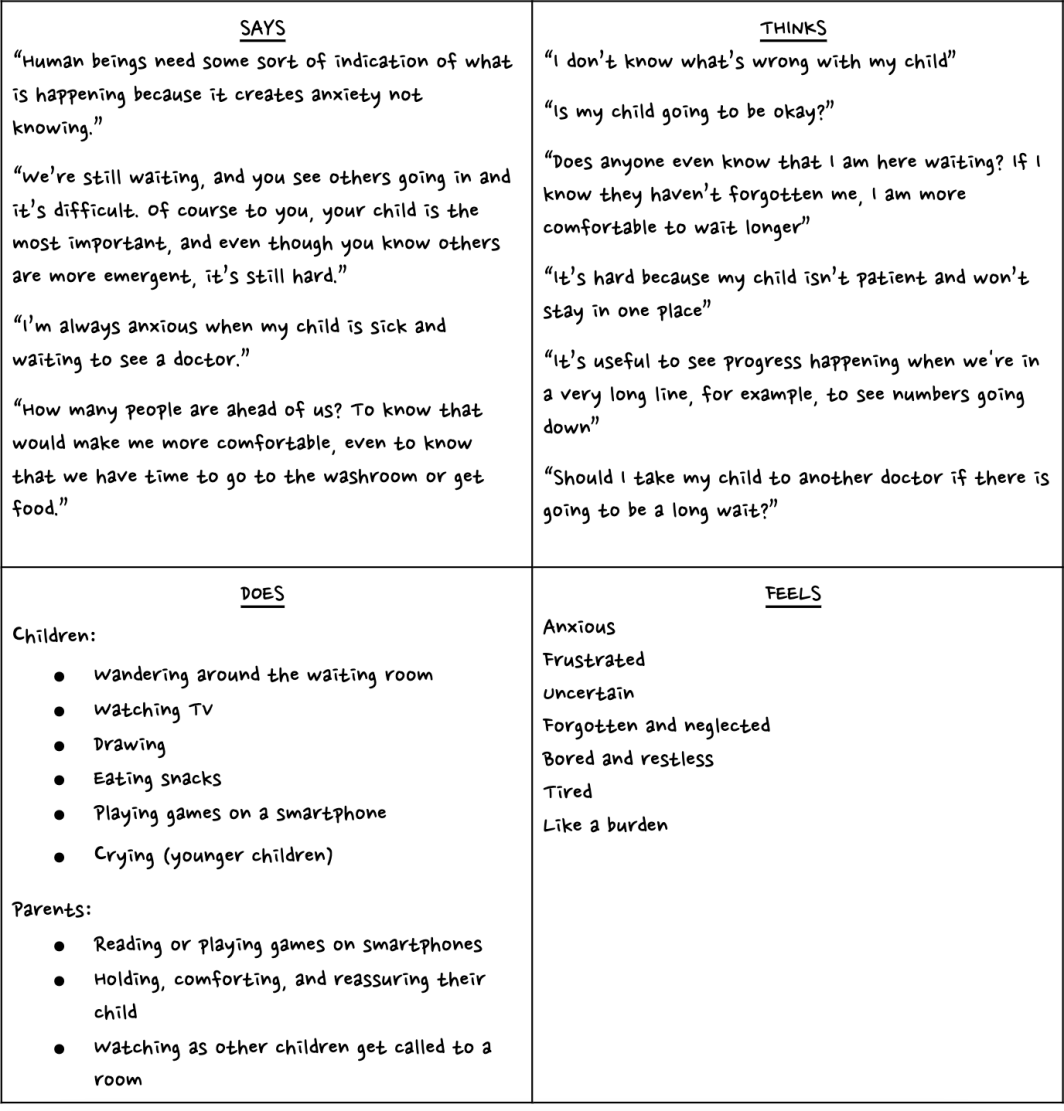
Empathy map.

Qualitative data from semistructured interviews and observations were analyzed independently by using a thematic analysis approach. Thematic analysis was conducted following Braun and Clarke’s 6-phase approach [22]: familiarization, coding, theme generation, reviewing themes, defining and naming themes, and reporting. First, transcripts were read independently by three members of the research team (SL, MM, ISK) for familiarization. Initial codes were generated independently and then combined to discover other codes. Differences in codes were reviewed and resolved item by item by the three research team members to ensure consistency. Codes were then grouped into potential themes based on patterns in the data. Themes were reviewed by the three members of the research team during team meetings. Data analysis was conducted iteratively, allowing for refinement as new insights emerged from ongoing interviews. Qualitative data were visually displayed in an empathy map (Figure 3). Data analysis and reporting were guided by the Consolidated criteria for reporting qualitative studies (COREQ) [23].

To ensure the rigor and trustworthiness of the study, we adhered to the criteria of credibility, transferability, and confirmability [24]. Credibility was established through repeated rereading of the data and regular research meetings to discuss the data and by including new questions in ongoing interviews to validate findings with new participants. Transferability was supported by providing detailed descriptions of the research context, participant demographics, and thematic findings so readers can assess the applicability of the results to other settings. Confirmability was maintained through reflexivity, where researchers met to discuss their prior assumptions and potential biases prior to starting the study and as the data were collected and analyzed. These strategies ensured that the findings were grounded in the data and not influenced by researcher bias.

## Results

### Overview

A total of 67 parents were approached to participate and 64 enrolled in the study (96%). Semistructured interviews were conducted with 64 parents (40 in round 1 and 24 in round 2). In addition, 5 interviews were conducted with triage nurses. Of the 64 parent interviews, 63 were conducted in English and 1 interview was conducted in Portuguese using a telephone interpretation service. All interviews took place on a weekday and occurred when the parent had waited less than 2 hours. Most parents were English-speaking and had a college or higher level of education. All parents owned a smartphone. Most children were under 10 years old and had previously visited the ED (see Table 2 for demographic data).

**Table 2.** Demographic data (N=64).

	Values
Time of day of interview (HH:MM), median (IQR)	13:55 (12:12-15:00)
Wait time at interview (hours), median (IQR)	1.0 (0.67‐1.63)
Relationship to child, n (%)	Mother: 38 (59) Father: 22 (35) Other (eg, guardian, grandparent): 4 (6)
Parent’s age (years), median (IQR)	40 (37‐44)
Parent’s education level, n (%)	Graduate/professional degree: 18 (28) College/university: 37 (58) High school: 7 (11) Other: 2 (3)
Parent’s preferred language, n (%)	English: 51 (80) Tamil: 2 (3) Urdu: 2 (3) Spanish: 2 (3) Bengali: 1 (2) Farsi: 1 (2) Hindi: 1 (2) Persian: 1 (2) Tagalog: 1 (2) Portuguese: 1 (2) Punjabi: 1 (2)
Parent has a smartphone (yes), n (%)	64 (100)
Parent uses apps on their smartphone (yes), n (%)	62 (97)
Child’s age (years), n (%)	<1 year old: 3 (5) 1‐5 years old: 26 (41) 6‐10 years old: 22 (34) 11‐18 years old: 13 (20)
Child’s total number of ED^a^ visits, median (IQR)	4 (2 to >5)

a ED: emergency department.

### Qualitative Analysis

Five key themes emerged from the qualitative analysis, from which the research team developed design criteria and solutions (Table 3). First, parents expressed significant anxiety due to the uncertainty surrounding their child’s health condition and the lack of clarity about how sick their child might be. Second, although parents could see health care staff moving throughout the ED, they frequently reported feeling forgotten when long periods passed without any updates. Third, parents demonstrated varying levels of health literacy, which was influenced by factors such as educational background, cultural context, preferred language, and previous health care experiences. Fourth, a lack of education about the ED process contributed to confusion about triage and wait times. Parents often did not understand why some children were seen more quickly than others. Finally, parents described an absence of indicators showing where their child was in the queue, which contributed to a sense of stagnation and frustration during their wait.

**Table 3.** Themes, design criteria, and solutions.

Theme	Supporting quotations	Design criteria	Solutions
Anxiety due to uncertainty: parents have significant feelings of anxiety due the to uncertainty of waiting with their child in the ED^a^.	“I’m always anxious when [my] child is sick and waiting to see a doctor.” “Human beings need some sort of indication of what is happening because it creates anxiety not knowing.” “My child is a little anxious because she doesn’t know what’s to come.”	The app must reduce uncertainty by giving parents information they can rely on.	The app will give parents more control over their time by providing their child’s personalized wait time.
Feeling forgotten: parents feel neglected and forgotten in the busy rush of the ED.	“I felt like an idiot asking for information.” “I felt like a burden.” “Knowing that people know we’re here would make us more comfortable to wait longer.”	The app must help make parents feel confident that they are being cared for.	The app will be available to parents throughout their stay so information is available when they want it.
Health literacy: parents report uncertainty about the severity of their child’s health issue and what is the most appropriate place to seek care.	“Is my child going to be okay? I don’t know necessarily how bad his condition is.” “I want to know if my child needs to be in the ED. We don’t want to wait 12 hours for a doctor to see her and say to take Tylenol, which is something we can do at home. Or if she doesn’t need to be here but go see her pediatrician.” “A nurse coming up to check on my kid early on during the wait to give us a scope of how severe [her health condition] is, some information about waiting and knowing what’s coming next was helpful.”	The app must provide medical education to guide parents about their child’s health issue.	The app will be designed to provide parents with relevant, personalized health information to read and watch as they wait.
Understanding the ED process: parents want to better understand the ED process, including the timing and order of steps in their journey.	“Last time I waited 12 hours, no one came to check up on us and I couldn’t get food for my child or myself.” “How many people are ahead of us, to know that would make us more comfortable to know what we have time for (the washroom or to get food).”	The app must give information to make the process of the ED more transparent.	The app will include a journey map that was cocreated with ED staff and parents. The journey map will have information about the steps in the ED process; the location of washrooms, food and drinks, prayer and reflection spaces, and breastfeeding rooms; and other important wayfinding information.
Indication of progress: parents perceive a lack of progress while sitting in the waiting room.	“We want to know the progress of our child in the queue.” “How many doctors are working? I want to know if the doctors are in the ED and not in a clinic somewhere.”	The app must provide parents with a sense of progress.	The app will show parents their child’s spot in the queue. This number will decrease as their child moves up in the queue to show progress.

a ED: emergency department.

### Tool Development

We developed a refined prototype through an iterative process of prototyping, software development, and redesign based on participant feedback and end user testing. Initially, a low-fidelity prototype was developed based on user needs identified during the thematic analysis. This prototype was tested with 24 participants in a controlled environment (in the ED waiting room with a research assistant observing), which allowed for the identification of usability issues and areas for improvement. Participants appreciated information about their child’s wait time and were empowered by the idea of knowing what to expect so they could plan their time. Participants made suggestions to include an audio component to the alerts, for example, a sound notification that their child was next to see a physician. Parents requested an inbox or chat feature so they could ask the health care team specific questions and receive personalized health information. Finally, parents wanted to be able to access wait time information from home so they could determine the optimal time to bring their child to the ED. Feedback from these sessions led to a series of redesigns, focusing on optimizing the data provided to parents, the user interface, and the interaction flow, based on observed participant behaviors and preferences (see Figure 2 for early prototypes). A point of disagreement emerged between triage nurses and parents. Triage nurses recommended including the data point “How long have we been waiting?” so they could refer to the app for objective data when parents expressed dissatisfaction about long wait times. Parents, however, found this feature unnecessary and even offensive to assume they might not know how long they had been waiting.

### AI Integration

In final stages of usability testing, the app was made available more widely and offered to all parents who were waiting in the ED. Parents accessed the app via a QR code on posters in the waiting room. Initially, the app showed all users the longest current wait time in the ED. In parallel with the user experience research, the AI component was developed to generate predictive and personalized wait time estimates, enabling parents to access wait time predictions and relevant health information tailored to their child’s presenting concern. The team developed machine learning and natural language processing algorithms to analyze free-text notes from the electronic medical record. The machine learning model analyzed several patient-specific and ED flow-related metrics to predict individual wait times. Patients were grouped into different categories based on what priority they would be expected to be assessed by a physician. The model analyzed key words from the triage note (eg, injury or fever) to deliver symptom-specific education. Metrics such as acknowledgment rates for education-based alerts and ED process-based alerts were tracked. The tool was tested in a validation phase for several months prior to being fully deployed. As of February 10, 2025, more than 7000 parents have used the tool.

## Discussion

### Principal Findings

The potential for technological innovations is rapidly evolving in health care. Many organizations have started to incorporate AI-powered tools into clinical practice. The use of AI to provide patients and families with individualized information has the potential to be highly beneficial. We propose a human-centered design methodology to ensure the needs of patients and families inform the design of the tools developed.

### Comparison With Prior Work

Our findings align with previous qualitative studies examining the ED wait time experience for patients and families. In pediatric EDs, parents often lack a clear understanding of ED operations, triage and waiting in particular, which leads to distrust in the system. Many parents perceive their child’s condition to be more urgent than other children’s, which can create frustration and dissatisfaction. Extended wait times may prompt some parents to consider leaving the ED before being seen under the assumption that a “true emergency” would have warranted faster care [25]. Prolonged wait times and insufficient communication can negatively impact perceived quality of care and health outcomes [7].

Existing literature strongly supports providing wait time information to patients and families. Access to wait times helps families manage other responsibilities and fosters a greater sense of control during a stressful experience [5,8]. In transportation research, the presence of wait time displays led individuals to perceive shorter wait times, even if actual wait times remained unchanged, demonstrating the power of perceived experience [26]. Prediction models using AI have begun to explore how to improve patient care by better communicating information about wait times and processes to patients and families [11,27]. Despite these potential benefits, a recent scoping review revealed that only 9.3% of Canadian EDs currently offer public-facing wait time displays [9]. There are no data about how many EDs offer personalized, predicted wait time data or how they communicate this information to patients and families.

AI tools and systems can be developed to solve long-standing challenges in health care. AI systems can be tailored to meet diverse needs, offering features like multilingual support, culturally appropriate messaging, and accessibility options such as large text, video sign language, or voice-to-text software [9]. A critical gap remains in the development and implementation of AI technologies in clinical spaces: the human-centered design process. To be effective and widely adopted, these innovations must be grounded in clinical need, co-designed with patients and families, and tested with patients, families, and health care providers to ensure they are accessible, useful, and safe.

### Principal Results

Participants in the study had wait times under 2 hours, which is typical for the overall lower patient volumes in the summer months. Most parents were English speaking, college educated, and all owned a smartphone. Most children were under 10 years old and had previous visits to the ED. Qualitative analysis of interviews and observations revealed 5 key themes that informed the development of the tool: (1) anxiety due to uncertainty, (2) feeling forgotten, (3) low health literacy, (4) not understanding the ED process, and (5) no indication of progress (see Table 3). Our exploration of the patient and parent experience informed the development of an AI tool that provides patients with personalized, predicted wait time and education information while they are in the ED. Prototype testing was crucial in refining the tool, ensuring that it was responsive to parents’ needs. Initial prototype testing with interviews and wireframes guided initial design directions before launching the app to all parents in the ED. The AI component of the tool was tested in a validation phase for several months prior to being fully deployed. The tool is now available to all families who come to the ED with their child.

### Limitations

There were some limitations to our data collection that should be noted. Interviews were conducted in the summer during daytime hours on weekdays due to research assistant availability. Our findings may not fully capture the heightened frustration and stress that parents experience during the longest wait times, which are most often at night, on weekends, and through the winter months. As a result, the study may not reflect the full range of patient experiences. However, we suspect the results would be even more dramatic if we had included more participants with longer wait times, as they would report more frustration, anxiety, and lack of transparency.

### Conclusions

This study used a human-centered design approach to explore parents’ experience in a pediatric ED to develop an AI tool to improve the waiting experience. We found that it was feasible to collect information from parents and families in the waiting room about their experience. In fact, 96% (64/67) of parents we approached were eager to participate. Parents described the anxiety and frustration associated with waiting with their child in the ED and were grateful for any information about the wait time or process. Key factors that contributed to parental anxiety and frustration during long wait times included a lack of information about the ED process, a perceived lack of progress, and uncertainty about the urgency of their child’s health issue. Receiving wait time and educational information that was personalized to their child was very appealing to parents. A key methodology in this study was the use of human-centered design to incorporate parents’ insights and experiences in the development of the AI tool.

The study highlights the significance of engaging with and understanding the user’s perspective in developing health care technologies. By prioritizing the parents’ experiences and insights, we have created a solution that addresses the challenge of communicating wait times and contributes to a more compassionate and efficient health care environment in the ED. The next step of this study is to make the tool more accessible by adding translations to other common languages and purposefully testing the tool with specific users, such as those who have low literacy, health knowledge, and access to technology. Further research examining the impacts of the tool on repeated use of the ED, health outcomes, efficiency, and cost savings would be valuable.

The integration of AI and human-centered design in health care extends beyond the pediatric ED and has broad implications for improving patient care, efficiency, and provider workflow across various clinical settings. AI-driven tools have the potential to support triage, optimize resource allocation, and provide real-time patient updates, potentially reducing wait times and improving patient satisfaction in EDs, outpatient clinics, and inpatient units. Human-centered design ensures these technologies are intuitive, accessible, and aligned with user needs, fostering better adoption by both patients and health care providers. However, widespread implementation comes with challenges, including variability in digital infrastructure across health care systems, concerns about data privacy, and the need for carefully designed systems to avoid perpetuating biases present in health care data. Despite these challenges, the study’s approach highlights the potential of combining AI with human-centered design to create scalable, patient-focused innovations that improve health care delivery across diverse settings.

## Supplementary material

10.2196/66644 Multimedia Appendix 1 Semistructured interview and usability testing guide.

10.2196/66644 Multimedia Appendix 2 Structured observation tool (AEIOU [Activities, Environments, Interactions, Objects, Users]).

## Abbreviations

AI: artificial intelligence
COREQ: Consolidated Criteria for Reporting Qualitative Studies
ED: emergency department
