# Supplementary material for: Creating a Parent-Informed Pediatric Emergency Department Wait Time App: Human-Centered Design Approach to Creating an AI Health Care Tool
Source: J Particip Med. 2025 Aug 29;17:e66644. doi: 10.2196/66644 (PMC12396731; doi:10.2196/66644)
Supplement: Multimedia Appendix 1 [file jopm-v17-e66644-s001.docx]

Appendix 1. Semi-structured interview and usability testing guide

Interview Guide:

1. How do you feel while waiting in the emergency department?

Probe: Why do you feel that way?

What is something that happened while waiting that was helpful?

What is something that happened while waiting that made it harder or more frustrating?

1. What other places outside a hospital setting have you visited where you had to wait? (e.g., service centre, theme park, restaurant)?

Probe: Which places were good waiting experiences and which were bad? Why?

1. Which of these places had a way to give information about your wait time (app, website, tickets, wait time board, other technology)?

Probe: What worked well? What didn’t work?

1. What information is useful for you to decide whether to keep waiting in the emergency department for your child’s health issue?

Probe: When would you like to get this information? (e.g. from home, at triage, in the waiting room)

1. Children are not always seen in the order they come to the ED. Doctors have to see children who are the most sick first. Does it help reduce your frustration to know if the children waiting ahead of your child are more sick?
2. How long do you think a child should have to wait to be seen by a doctor when they come to the emergency department?

Probe: What if the ED is very busy?

1. If we could tell you approximately how long your wait will be today, for example, 1 hour, would it be reasonable for us to tell you your wait would be 1 hour and 10 minutes, 1 hour and 30 minutes, 1-2 hours?
2. When you entered the emergency department, did you notice the wait time screen with information about the wait time? (yes/no)

Probe (if yes): Did you find the wait time screen useful? Why?

(if no): What information would be useful on the screen? What would you change about the information/location of the board?

Would it be more useful if it was the wait time specifically for your child?

1. Do you have any other questions?

Interview and Usability Testing Guide:

1. While waiting in the ED, what information do you wish you knew about how the ED works?

Probe: e.g., directions, water, food, washroom, information about their child’s medical condition, the process in the emergency department (do you know where the nearest bathroom is or the nearest water fountain, where they can read about medical information, using interpreters)

1. What questions do you wish you could ask while waiting?

Probe: Who would you ask these questions to? Who would be the best person to answer them?

1. What questions do you have about your child’s health issue?

Show a screenshot on a phone or log into the parent’s phone to show them

1. What do you think of this app?

Probe: what do you like the best? What do you not like?

1. Do you think this app could help families understand how long they might wait?
2. Is there anything that doesn’t make sense to you?
3. Can you tell me what the words and descriptions on the page mean? (parent explains)

Probe: (if unable or wrong)which aspects of this are confusing?

1. How can we change the words/rephrase to make it make more sense?
2. Can you explain what the speedometer is trying to show?

Probe: (if able) Does it help you understand how busy it is in the emergency department? (Y/N)

Probe: (if unable or no) is there a better way to visualize the wait time information?

1. How can we change the words/rephrase to make it make more sense?
2. What do you think of the layout (where words and images are on the screen)?

Probe: How would you change the layout to make it better?

1. Do you like the colours used in the app?

Probe: (if no): which colours would be better?

1. Is there anything you can suggest to make this better?
2. Do you have any other comments?
