# Supplementary material for: Creating a Parent-Informed Pediatric Emergency Department Wait Time App: Human-Centered Design Approach to Creating an AI Health Care Tool
Source: J Particip Med. 2025 Aug 29;17:e66644. doi: 10.2196/66644 (PMC12396731; doi:10.2196/66644)
Supplement: Multimedia Appendix 2 [file jopm-v17-e66644-s002.docx]

Appendix 2. Structured observation tool (AEIOU)

| Activities: What actions and behaviors are people taking to reach goals? |
| --- |
| Environments: What is the overall setting in which the activities are taking place? How are people behaving in the environment? |
| Interactions: What are the basic interactions occurring for people to reach goals? What effect do people have on activities and environment? |
| Objects: What are all the details that form the environment? How do objects relate to people, activities and interactions? |
| Users: Who are the people being observed? What are their personalities like? How do they engage with other people to reach goals? |
| Extreme Users: Describe people who have been waiting a long time/no time at all. Who seem have an easy time waiting/very hard time & why. Who seem to understand the system/who don’t. |

Fitzpatrick D. AEIOU observation framework. Open Practice Library. Nov 30, 2018. URL: <https://openpracticelibrary.com/practice/aeiou-observation-framework/>
